# Locus Coeruleus Neurons’ Firing Pattern Is Regulated by ERG Voltage-Gated K^+^ Channels

**DOI:** 10.3390/ijms232315334

**Published:** 2022-12-05

**Authors:** Sonia Hasan, Francis Delicata, Leonardo Guasti, Claudia Duranti, Fatemah Mousalem Haidar, Annarosa Arcangeli, Paola Imbrici, Mauro Pessia, Mario Valentino, Maria Cristina D’Adamo

**Affiliations:** 1 Department of Physiology, Faculty of Medicine, Kuwait University, Safat 13110, Kuwait; 2 College of Pharmacy, Rady Faculty of Health Sciences, University of Manitoba, Winnipeg, MB R3E 0T5, Canada; 3 Centre for Endocrinology, William Harvey Research Institute, Faculty of Medicine and Dentistry, Queen Mary University of London, London EC1M 6BQ, UK; 4 Department of Experimental and Clinical Medicine, Section of Internal Medicine, University of Florence, 50121 Firenze, Italy; 5 Department of Physiology, College of Medicine and Health Sciences, United Arab Emirates University, Al Ain 17666, United Arab Emirates; 6 Department of Pharmacy—Drug Sciences, University of Bari “Aldo Moro”, 70121 Bari, Italy; 7 Department of Physiology & Biochemistry, Faculty of Medicine & Surgery, University of Malta, MSD 2080 Msida, Malta; 8 Department of Medicine & Surgery, LUM University “Giuseppe Degennaro”, Casamassima, 70010 Bari, Italy

**Keywords:** locus coeruleus neurons, noradrenergic system, ERG K^+^ channels, *ether-à-go-go–related gene*, WAY-123,398, class III anti-arrhythmic drug

## Abstract

Locus coeruleus (LC) neurons, with their extensive innervations throughout the brain, control a broad range of physiological processes. Several ion channels have been characterized in LC neurons that control intrinsic membrane properties and excitability. However, ERG (*ether-à-go-go–related gene*) K^+^ channels that are particularly important in setting neuronal firing rhythms and automaticity have not as yet been discovered in the LC. Moreover, the neurophysiological and pathophysiological roles of ERG channels in the brain remain unclear despite their expression in several structures. By performing immunohistochemical investigations, we found that ERG-1A, ERG-1B, ERG-2 and ERG-3 are highly expressed in the LC neurons of mice. To examine the functional role of ERG channels, current-clamp recordings were performed on mouse LC neurons in brain slices under visual control. ERG channel blockade by WAY-123,398, a class III anti-arrhythmic agent, increased the spontaneous firing activity and discharge irregularity of LC neurons. Here, we have shown the presence of distinct ERG channel subunits in the LC which play an imperative role in modulating neuronal discharge patterns. Thus, we propose that ERG channels are important players behind the changes in, and/or maintenance of, LC firing patterns that are implicated in the generation of different behaviors and in several disorders.

## 1. Introduction

Located in the anterior pons, the locus coeruleus (LC), with its extensive innervations throughout the central nervous system, is the major noradrenergic nucleus of the brain and thereby controls a broad range of physiological processes that include cognition, learning and memory, sleep–wake cycle, arousal, attention, mood, anxiety and pain [1,2,3,4,5,6,7]. These and other neurophysiological processes are known to be affected by changes in the discharge properties of LC neurons [7], which in turn are dependent on the intrinsic membrane properties and the ion channel constituents of these neurons. For example, in a previous study, we demonstrated that the inwardly rectifying potassium channel Kir5.1 plays a crucial role in defining the CO_2_/pH sensitivity of LC neurons. The LC is a CO_2_-chemosensitive region of the pons where more than 80% of its neurons respond to hypercapnic acidosis with an increase in firing rate [8,9]. Kir5.1 is likely a key contributor to LC’s firing response to hypercapnic acidosis [8].

LC neurons are endogenous pacemakers that fire spontaneous and repetitive action potentials [9]. Besides Kir5.1, several ion channels have been characterized in LC neurons [8,10,11,12,13,14,15]. However, ERG (*ether-à-go-go–related gene*) channels that belong to the KCNH super-family of K^+^ channels [16], and that are particularly important in setting neuronal firing rhythms and automaticity [16,17,18], have not been identified thus far in the LC. ERG channels can be subdivided into three groups: ERG-1 (*KCNH2*, Kv11.1), ERG-2 (*KCNH6*, Kv11.2) and ERG-3 (*KCNH7*, Kv11.3). In addition, a long splice variant of ERG-1 (ERG-1A) and a short one (ERG-1B) have been identified [19]. ERG channels have been implicated in the regulation of excitability, discharge pattern, spike-frequency adaptation and the resonance properties of neurons [17,18,20,21].

The human ERG-1 (hERG-1; *KCNH2*) encodes the pore-forming subunit of a rapid-delayed rectifier K^+^ channel, the current (I_Kr_) through which ensures a fast repolarization phase of the cardiac action potentials and, consequently, regulates heart rhythms [22]. Its dysfunction is known to cause *long QT syndrome* and inherited and acquired cardiac arrhythmias [22,23]. However, while the role of this channel is quite well-established in cardiomyocytes, in the central nervous system its neurophysiological and pathophysiological roles remain unclear despite its expression in several crucial brain structures [18,19,20,24,25,26,27,28].

By performing immunohistochemical and electrophysiological investigations, we found that ERG-1A, ERG-1B, ERG-2 and ERG-3 are highly expressed in LC neurons, where members of the ERG channel family modulate intrinsic electrical properties and rhythmic firing.

## 2. Results

### 2.1. Immunohistochemical Localization of ERG Channels within Murine LC Nuclei

**Figure 1:**
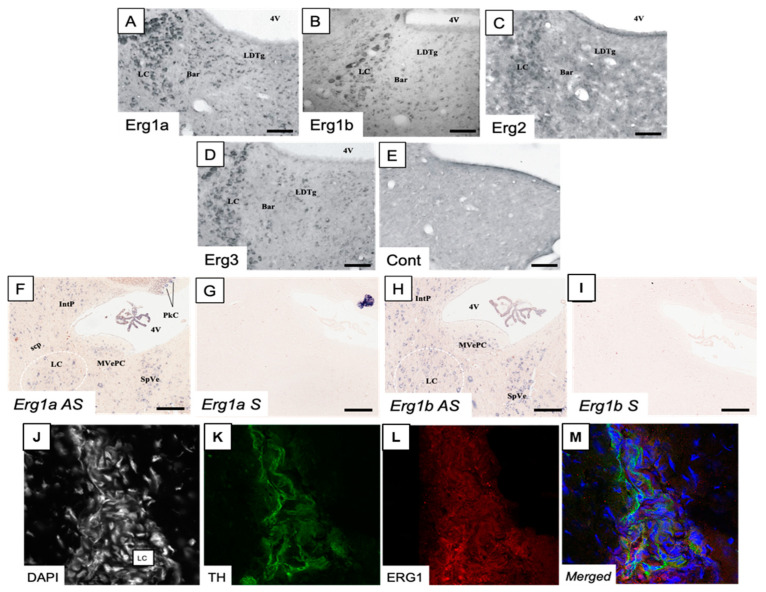
Expression of ERG1 channels in LC neurons. (**A**–**D**) Expression of Erg1a (**A**), Erg1b (**B**), Erg2 (**C**) and Erg3 (**D**) proteins in the LC and adjacent areas, as visualized through immunohistochemistry. (**E**) represents a stained section where the primary antibody was omitted. (**F**–**I**) Expression of *Erg1a* (**F**) and *Erg1b* (**H**) mRNAs in the LC and adjacent areas (sagittal sections), via non-radioactive in situ hybridization using anti-sense probes. Sections processed in parallel with sense probes (**G**,**I**) showed no signal. High magnification images of LC stained with the anti-TH (**K**) and anti-ERG1 (**L**) antibodies. Images of neurons stained with DAPI (**J**) and merged (**M**) are reported. Note that cells within the LC nucleus (indicated as LC) were heavily stained by the anti-TH specific antibody (green signal). Staining with anti-ERG1 antibody (red signal) was also present. Scale bars (**A**–**I**) = 100 μm, (**J**–**M**) image size is 185 × 185 μm. Abbreviations: 4V, 4th ventricle; Bar, Barrington’s nucleus; IntP, interposed cerebellar nucleus, posterior part; LC, Locus coeruleus; LDTg, laterodorsal tegmental nucleus; MVePC, medial vestibular nucleus, parvicellular part; PkC, Purkinje cells; SpVe, spinal vestibular nucleus.

In order to determine the localization of ERG channel types within the LC nuclei, rabbit polyclonal anti-ERG-1A, anti-ERG-1B, anti-ERG-2 and anti-ERG-3 antibodies were produced, purified and tested as previously described [19]. Using these antibodies, positive immunoreactivity was observed within the brainstem slices that were dissected from C57BL/6J mice. LC neurons were stained with the anti-ERG-1A, anti-ERG-1B, anti-ERG-2 and anti-ERG-3 antibodies and the signal was highly specific (Figure 1A–E). Notably, the images point out that numerous cell bodies located within the LC nucleus were immunopositive for all channel types. As reported for other brain regions [19,20], ERG-2 appeared to be the least expressed among the channel subfamily. For ERG-1A and ERG1-B, expression in the LC was also confirmed via in situ hybridization (Figure 1F–I). Moreover, ERG-positive cells were Tyrosine Hydroxylase (TH) positive, further confirming their expression in LC neurons (Figure 1J–M). Overall, these observations demonstrated that ERG-1A, ERG-1B, ERG-2 and ERG-3 channel types are expressed by LC neurons.

### 2.2. ERG Channels Regulate the Spontaneous Activity of LC Neurons

**Figure 2:**
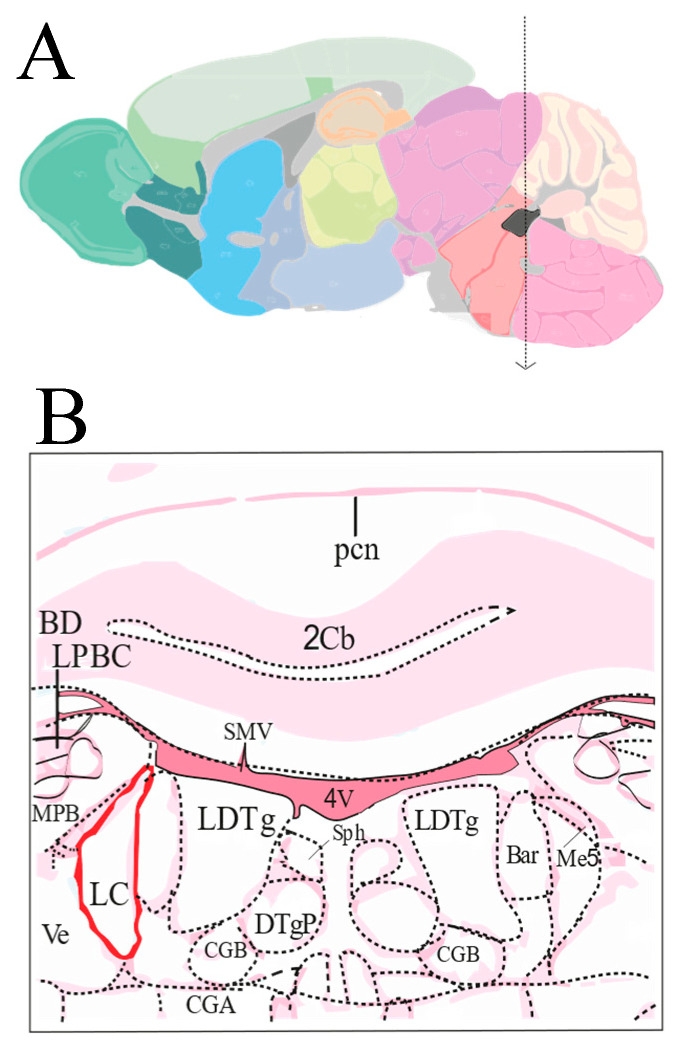
Anatomical localization of LC nucleus. (**A**) Sagittal section of the murine whole brain, each color distinguishes different regions in the brain, with black showing the localization of the LC nucleus in the pons of brainstem. (**B**) Cartoon showing the boundaries of the LC nucleus in red at the level of the Vth ventricle, compared to other surrounding structures in pink, where sections were dissected.

**Figure 3:**
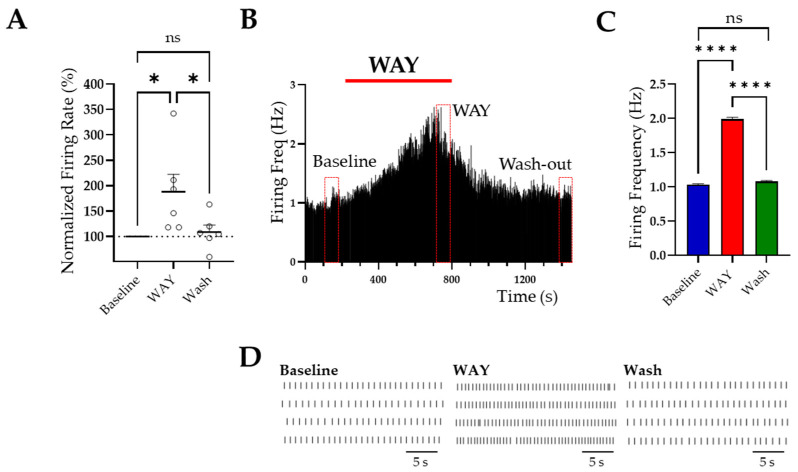
WAY increased LC neuron firing frequency. (**A**) Plot showing LC neuron firing rate (%) increased after WAY application and recovered back to baseline levels during the wash-out period. One-way ANOVA was performed on the mean of data points taken from a 60 s period representing the peak effect of each condition. * *p* < 0.05; *n* = 6 neurons each from a different mouse. (**B**) Firing frequency from a representative LC neuron. The firing frequency of a 60 s period in control/baseline conditions, during WAY application and during the wash-out. (**C**) Bar graph showing data from the neuron in (**B**). One-way ANOVA was performed on the mean data points enclosed by the red rectangles shown in (**B**); **** *p* < 0.0001. (**D**) Raster plots of the data shown in (**B**).

**Figure 4:**
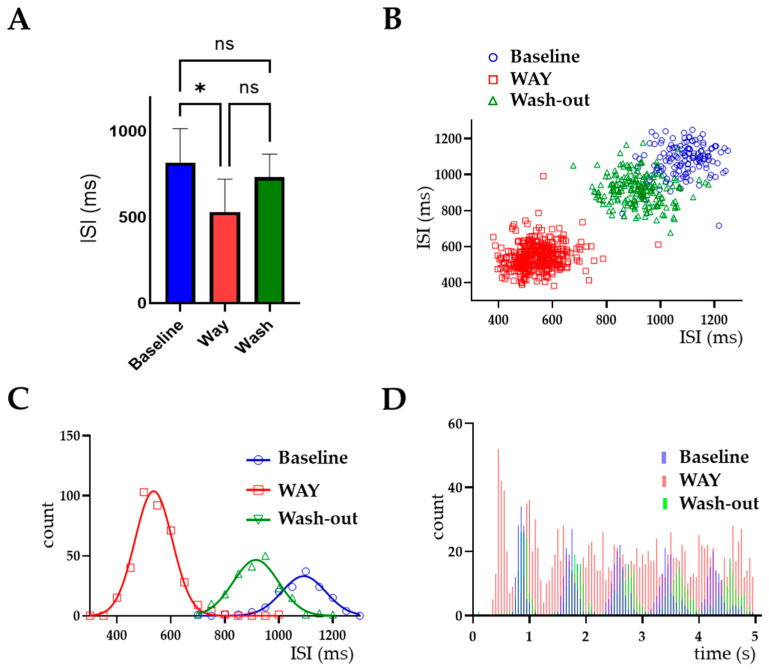
WAY alters LC neuronal firing pattern. (**A**) Bar graph showing a decrease in mean inter-spike interval (ISI) with WAY application (* *p* < 0.05, ns = no significant difference, *n* = 6), that was reversed by wash-out. (**B**–**D**) Representative data from a single neuron, showing ISI scatterplots (**B**), ISI distributions fitted with the Gaussian equation Y = Amplitude × exp(−0.5 × ((X-Mean)/SD)^2^) (**C**) and ISI auto-correlograms (**D**) calculated in baseline conditions (blue), during WAY application (orange) and after drug wash-out (green), from a 200 s representative period of a single recording. Raw ISI values are plotted. The plots point out a regular firing pattern in the baseline and wash-out periods that is remarkably evidenced by the regular peaks in the ISI auto-correlograms. By contrast, a more irregular firing pattern of LC neurons during WAY application was clearly seen by the less defined peaks.

LC neurons in the pons were confined to the border of the IVth ventricle (Figure 2A,B), within an anatomical area that was readily identifiable for patch-clamp recordings from living brain slices imaged under infrared differential interference contrast microscopy. Moreover, the electrical properties of LC neurons recorded in brain slices were remarkably uniform [8], which further facilitated their recognition and recording. Slices were dissected from P40 ± 10-day-old mice and the electrical properties of LC neurons were assessed by means of whole-cell patch-clamp recordings in current-clamp mode [8]. LC neurons were spontaneously active (within the range of 0.5–5 Hz), displayed pacemaker-like firing, and possessed consistent action potential parameters. In particular, their resting membrane potentials oscillated between −32 mV and −55 mV, displayed input resistance of 366 ± 41 MΩ (*n* =12) and basal firing frequencies of 3.4 ± 0.5 Hz (*n* = 12). To assess the functional role of ERG channels in LC neurons, we used WAY-123,398 (WAY), an ERG channel blocker, the efficacy of which we had previously reported in brain-slice recordings [20,29,30,31]. The electrophysiological properties of LC neurons modulated by ERG channels were analyzed under control conditions by perfusing the brain slices with a CSF and adding 10 μM WAY (final concentration in aCSF) to investigate the effects of channel block on the spontaneous discharge of these neurons and during a wash-out period. An enhancement of the spontaneous firing frequency was observed in LC neurons after the addition of WAY (Figure 3A, Table 1; WAY 188 ± 35, % of baseline vs. wash-out 109 ± 13.6, % of baseline *p* < 0.05; *n* = 6 neurons). There was no significant difference between the wash-out and baseline levels, indicating that this enhancement was reversed during drug wash-out. Figure 3B shows the effect of WAY on a representative LC neuron. WAY was able to almost double the firing frequency and cause an neuronal firing irregularity, which consisted of frequency fluctuations with unpredictable intervals (Figure 4D, raster plot).

While there was no significant difference in the coefficient of variation (CV), the inter-spike interval (ISI) decreased following WAY application (baseline 819 ± 197 ms vs. WAY 531 ± 191 ms, one-way ANOVA, *p* < 0.05, *n* = 6, Figure 4A). Wash-out recovered the ISI back to 733 ± 135 ms, with no statistically significant difference between it and the baseline (*n* = 6, *p* = 0.6; Figure 4A, Table 1). The regular firing pattern of LC neurons recorded in control conditions was characterized by the well-defined cluster in the scatterplot (Figure 4B), the narrow bell-shaped distribution of the ISI histogram (Figure 4C) and the well-defined multiple peaks in the auto-correlogram (Figure 4D). The discharge irregularity brought about by WAY was demonstrated by the more dispersed cluster in the scatterplot (Figure 4B), and the flatter distribution with fewer peaks in the ISI auto-correlogram (Figure 4D).

## 3. Discussion

In this study, and for the first time, proof of the presence of ERG-1A, ERG-1B, ERG-2 and ERG-3 channel subunits in the LC was established. The widespread expression of ERG channels in the brain and in very critical regions, such as the LC, indicates the importance of this channel type in the regulation of CNS functions. The study, also for the first time, brings forth the channel’s imperative role in regulating neuronal discharge patterns in LC neurons. Specifically, we reported in LC neurons a significant enhancement of spontaneous tonic firing frequency accompanied by an increase in firing irregularities after specific block of the ERG channels, indicating that ERG channels are a mechanistic tool that prevents increased firing rates and discharge irregularities in LC neurons.

Previous studies on other brain regions have shown that ERG channels variably affect neuronal excitability and firing frequency. ERG channel blockers increase [17,18,20,26] or decrease [32] firing frequency. They may cause or enhance spike-frequency adaptation [26], reduce spike-frequency adaptation, converting it to regular firing [17,18,20], or affect the resonance properties of neurons [20]. The variation may be due to differences in the characteristics of the areas and dynamic properties of the neurons, including the type of inputs impinging on the neuron and the resulting intracellular signaling cascades. Indeed, ERG channels are modulated by several intracellular signaling pathways that may mediate their role as regulators of excitability. In particular, protein kinase A and C inhibit ERG channel function by direct ERG subunit phosphorylation, suggesting that ERG channels, and, consequently, neuronal excitability, can be regulated by a variety of G-protein coupled receptors [33]. Notably, the stimulation of M_1_ and M_2_ muscarinic receptors activates PKC, and raises [Ca^2+^]_i_ and inhibits human ERG currents [34,35]. In the LC, acetylcholine induces increases in the neuronal firing rate, which is antagonized by the M_1_ antagonist pirenzepine [36,37]. Similarly, activation of the metabotropic glutamate receptor mGluR1 in Purkinje cells and mitral/tufted cells of the olfactory bulb reduced ERG currents and increased firing frequency and excitability [35,38]. Intriguingly, mGluR1 receptors are expressed in LC neurons [33], where they may play a role in the enhancement of firing rate via ERG channel modulation.

LC neurons are intrinsic pacemakers that fire spontaneously in the absence of synaptic input. Recent optogenetic studies have confirmed that there is a variability in the LC neuronal firing rate and pattern that mediates the diverse behavioral functions associated with the LC [7,38]. For example, the LC’s widespread innervation throughout the CNS allows for global brain arousal, and increases in LC neuronal firing rates are correlated with increased synchrony and arousal [39,40,41]. Moreover, different LC discharge rates are associated with different levels of arousal and anxiety states [7,39,40]. In the LC, increased tonic firing, such as that which we have observed with the ERG channel block, induces anxiety and aversive behaviors [42], whereas a decrease in LC tonic discharge rate is associated with a disengagement from the environment [6,43] and is evident, for instance, during slow-wave sleep. In contrast, there is discharge silence during REM sleep. During the arousal state, a moderate tonic firing is necessary for the occurrence of phasic burst firing in response to salient stimuli [44]. Unlike the 1–6 Hz continuous discharge in the tonic mode, in the phasic mode LC neurons synchronously fire in bursts of 10–15 Hz, permitting optimum performance and allowing for a shift in focus to task-related functions as well as to novel stimuli [6,45]. Excessive or insufficient tonic firing would impede phasic firing and its associated task performance. These electrophysiological observations indicate strongly that changes in the firing rate are involved in the transition between states of arousal and consciousness as well as moving from one stage of the sleep–wake cycle to another. While these findings bring forth LC firing patterns as a mechanism for the generation of different states and behaviors, we propose that ERG channels are important players behind the changes in, and/or maintenance of, firing rates. As such, it would be interesting to investigate ERG dysfunction in different anxiety, attention, and sleep disorders.

Many antipsychotic medications, such as *Haloperidol* and *Chlorpromazine*, are potent ERG channel inhibitors or blockers [16,46], indicating a possible association between altered ERG activity and cognitive dysfunction. The antipsychotic effects of these medications are induced by blocking dopamine receptors. However, these medications also block ERG channels at similar therapeutic concentrations, thereby affecting neuronal excitability [16,18,46]. As such, an ERG channel blockade may contribute to the antipsychotic effect. Notably, in a human study, *Haloperidol* administration significantly improved memory performance, which was associated with increased LC activity [47]. On the other hand, antipsychotics, as well as antibiotics and antiemetics, that are known to inhibit or block ERG channels, can predispose certain individuals to *long QT syndrome* and cardiac arrhythmias due to the blockade of cardiac ERG channels. However, LC noradrenergic activation increases the heart rate and the adverse risks associated with tachycardia by depressing the activity of parasympathetic cardiac vagal neurons [48]. We propose that these drugs’ blockage of the ERG channels in LC neurons alter their firing pattern and possibly change the heart rate, potentially enhancing the risk of cardiac arrhythmia. Thus, the potential role of ERG channels in the LC and the mechanisms of action of these medications would deserve further investigation.

There is evidence for the pathological role of ERG channels in the brain. The significant co-occurrence of epilepsy with *long QT syndrome* is noteworthy [49,50]. Furthermore, mutations in the *KCNH7* gene (ERG-3, Kv11.3) were associated with bipolar spectrum disorders [51,52]. The results of these studies have shed light on the possible role of ERG channel dysfunction in diseases involving the LC. The role of the LC in bringing about schizophrenia’s cognitive symptoms has been recently emphasized with the observation that positive symptoms are consistent with hyperactivity of the LC noradrenergic system, while negative symptoms are consistent with a hypoactivity of this system [53]. Interestingly, ERG dysfunction and its associated disruption in neural firing has been implicated in schizophrenia [54,55]. ERG3 expression in schizophrenic hippocampi was 2.5-fold greater than ERG-1A, resulting in a rapidly deactivating K^+^ current and a high-frequency, non-adapting firing pattern [54]. More importantly, an association between ERG mutations and schizophrenia has been established [54,55,56,57]. Finally, stress-initiated LC dysfunction has been proposed along with genetic susceptibility as being responsible for observed tonic- and phasic-firing imbalances that lead to schizophrenic dysfunctional network integration and cognitive deficits [54]. Are these schizophrenic LC firing imbalances and altered excitability a result of abnormal ERG currents? This question remains to be answered, and suggests a need for novel therapeutic investigation based on ERG-modulating agents.

LC’s role in the prodromal or premotor stage of Parkinson’s disease is well established [58]. In fact, LC cells show *αSyn* aggregation and *Lewy* pathology formation several years earlier than dopaminergic SN neurons’ degeneration is apparent. In Parkinson’s disease models, *αSyn* overexpression or *rotenone* exposure enhanced the spontaneous LC discharge frequency, which was associated with a marked decrease of after-hyperpolarization amplitude [58]. The small-conductance Ca^2+^-activated K^+^ (SK) channels were proposed as mediators of this enhancement. Interestingly, in Parkinsonian rats, ERG K^+^ channel blockers reduced burst discharges and the firing frequency of subthalamic nucleus neurons, which led to an improvement in locomotor deficits, while the activators led to an increased burst mode and impaired motor function in normal rats [21]. Here, we propose that ERG channels reduced the mediators of the spontaneous firing rate in the LC. Therefore, it would be interesting to explore LC ERG channel dysfunction as a possible contributor to Parkinson and, more importantly, to explore the modulation of the LC ERG channel as a therapeutic option.

In conclusion, we reported the protein expression of ERG channels in mice LC nuclei and we presented electrophysiological data to confirm not only that they are functional, but that they play a key role in the LC neuronal discharge pattern. We presented ERG channels as essential means for the control of speed and stability of LC firing. The outcome of ERG channel activity on LC neuronal excitability may be a contributing mechanism towards LC’s regulation of several physiological processes attributed to it. Furthermore, ERG K^+^ channel dysfunction may constitute an important pathophysiological mechanism for disorders of the central nervous system associated with LC and the noradrenergic system, and should be considered during pharmacotherapeutic interventions and vigilance. We anticipate that, in the near future, known neurological and psychiatric disorders will be shown to be largely attributed to ERG channel dysfunction in brain areas including the LC.

## 4. Materials and Methods

### 4.1. Immunohistochemistry

This study was carried out using brainstem tissue dissected from young (P10) and adult (P60) mice (C57BL/6J). This procedure was performed in accordance with national and international regulations, approved by the ethics committee, and compliant with ARRIVE guidelines. Mice were transcardially perfused with saline (0.9% *w*/*v* sodium chloride), followed by paraformaldehyde (Sigma, St. Louis, MO, USA; 4% *w*/*v*) in phosphate-buffered saline (PBS, 3.2 mM Na_2_HPO_4_, 0.5mM KH_2_PO_4_, 1.3 mM KCl, 135 mM NaCl, pH7.4) under terminal anesthesia induced by chloral hydrate (300 mg/kg *i.p.*). Brains were removed and post-fixed overnight at 4 °C in the same fixative, then soaked in 30% sucrose for cryoprotection. Brains were rapidly frozen and slices were obtained with a freezing cryostat at 30 μm thickness and collected in ice-cold PBS. Immunohistochemistry was performed as described previously [19]. The specificity of antibodies in recognizing ERG proteins in mouse brain-sections has been previously described [19]. Images were acquired with a Leica DMR light microscope equipped with a Leica DC200 digital camera, converted to greyscale, and adjusted for brightness and contrast using Adobe Photoshop (v.6.0; Abode Systems, San Jose, CA, USA). Each immunohistochemical experiment was repeated by using brainstem slices collected from three animals at both P10 and P60.

Immunofluorescence (IF) on cells was performed following the protocol previously described by Guasti et al., 2008 [19]. For IF on mice brains, the latter was kindly provided by Prof. Andrea Becchetti (Department of Biotechnology and Biosciences, University of Milano-Bicocca, Milano, Italy), snap frozen in liquid nitrogen and sectioned with a cryostat in 20 mm sections to localize the LC area. After 2 hours of blocking in PBS with 10% BSA, sections were incubated for a further 2 hours with anti-TH (Millipore) (diluted 1:10), followed by 1 hour incubation with antimouse Alexa Fluor 488 (Thermo Fisher Scientific). Incubation with poly-hERG1 was performed overnight (diluted 1:100) at 4 °C. Nuclei were stained with Hoechst (1:1000 in PBS, 45 minutes; Merck Sigma). Images were captured using a Nikon TE2000 confocal microscope, as described in Lottini, et al., 2021 [59].

### 4.2. Tight-Seal, Whole-Cell Recordings

This study was carried out using brainstem slices dissected from adult C57BL/6J male mice (P40 ± 10). Mice were decapitated after 30 min of deep chloral hydrate (4% in saline, intraperitoneal) anesthesia and the cranium was opened to expose the entire brain. The brain was rapidly removed and put into an ice-cold oxygenated solution of 2.5 mM KCl, 26.2 mM NaHCO_3_, 1 mM NaH_2_PO_4_, 2 mM MgSO_4_, 0.5 mM CaCl_2_, 11 mM D-glucose, 238 mM sucrose, saturated with 95% O_2_ and 5% CO_2_, at pH 7.4. Coronal slices (220 µm thickness) were cut from the brainstem (submerged in the same ice-cold solution) using a Vibratome. Slices containing the LC were incubated at 30 °C for 30 min in *artificial cerebrospinal fluid* (aCSF) (125 mM NaCl, 2.5 mM KCl, 26 mM NaHCO_3_, 1.25 mM NaH_2_PO_4_, 1 mM MgCl_2_, 2.4 mM CaCl_2_, 11 mM D-glucose, saturated with 95% O_2_ and 5% CO_2_, pH7.4) and were transferred to a recording chamber (500 µl volume). The slice was secured by means of a nylon mesh glued to a U-shaped platinum wire that totally submerged the tissue in a continuously flowing aCSF at a rate of 2.5 ml/min (warmed to 32 ± 1 °C). All neurons fired spontaneously at frequencies between 0.5 and 5 Hz (3.6 ± 1.1 Hz) when perfused with control aCSF (95% O_2_ and 5% CO_2_, pH7.4).

Patch-clamp recordings were performed from LC neurons under visual control (using Hamamatsu and Axioskop 2FS infrared optics) and were recorded in the current-clamp configuration using an EPC-9 amplifier and acquired with Patch Master software (HEKA Elektronik GmbH, Reutlingen, Germany). Patch glass pipettes (King Precision Glass, Claremont, CA, USA) were pulled in several stages to a tip with approximately 1 µm outside diameter, had resistances of 3–5 MΩ and were filled with an intracellular solution containing 115 mM CH_3_KO_4_S, 20 mM KCl, 1.5 mM MgCl_2_, 5 mM HEPES, 0.1 mM EGTA, 2 mM Mg-ATP, 0.5 Na-GTP and 10 mM C_4_H_10_N_3_O_5_P (pH 7.4). The liquid junction potential was calculated to be approximately 10 mV (pipette negative relative to bath). All data were obtained using this solution and left uncorrected. After the seal formation (2–10 GΩ), a whole-cell configuration was obtained by further suction of the patch membrane (200–300 MΩ). Current-clamp recordings were performed after ≥10 min of stable seal formation and were analyzed only if action potential amplitudes were ≥80mV, resting membrane potentials oscillated between −30 and −50 mV and series resistance changed <20% throughout the entire recording period.

### 4.3. ERG Channels’ Blockage

To assess the functional role of ERG channels in LC neurons, we used 10 μM WAY-123,398 (C_19_H_25_N_5_O_4_S) which belongs to the class III anti-arrhythmic agents that block all ERG channel types at this concentration, in a voltage-independent fashion [29,30]. WAY-123,398 was a generous gift from Dr. W. Spinelli, Wyeth-Ayerst Research, Princeton, NJ, USA. WAY-123,398 aliquots of stock solution (1mM) were prepared in distilled water and stored at −20 °C. The blocker was diluted to its final concentration in aCSF. A complete exchange of the bath solution in the recording chamber occurred in approximately 2 min.

### 4.4. Data Analysis and Statistical Evaluation

Action potential peaks were detected using a suitable voltage threshold on Clampfit v10.7 software (Molecular Devices, San Jose, CA, USA). The firing frequency, in Hz, and inter-spike intervals (ISI), in ms, were automatically extracted following peak detection. The coefficient of variation (CV), a measure of firing irregularity, was calculated as follows: CV = σ/μ. Firing regularity analysis was performed using a Spike2 software script (Cambridge Electronic Design, Cambridge, UK) developed by Dr. Massimo Pierucci: (a) ISI scatterplots were used to compare differences in consecutive ISI data points. A compact scatter indicated a regular firing pattern. (b) ISI histograms were prepared and visualized as a Gaussian least-square fit, to test for a normal or skewed distribution of the data points; the former indicated a regular firing pattern. (c) ISI auto-correlograms were used to test for regular correlated peaks that signified a regular firing pattern. All statistical analyses and graph plotting were performed with GraphPad Prism v9.1 (GraphPad software, CA, USA). A Kolmogorov–Smirnov test was carried out on each data set to test for a normal distribution of data. A parametric or non-parametric one-way ANOVA with multiple comparisons was subsequently performed to compare the means of datasets before and after drug application and during wash-out. Statistical tests were carried out using representative 60 s periods of the raw data. Data were presented as the mean ± SEM.

## Figures and Tables

**Table 1:** Effects of WAY application on firing properties and firing pattern of LC neurons.

WAY-123,398 10 μM (*n* = 6 Neurons)			Wash-Out (*n* = 6 Neurons)		
Firing Freq ^+^	ISI ^+^	CV ^+^	Firing Freq ^+^	ISI ^+^	CV ^+^
188 ± 35 *	62 ± 10 *	124 ± 14	109 ± 14 *	102 ± 14.4	112 ± 10

^+^ % of baseline ± SEM; percentage of variation in firing rate, ISI and CV during the application and wash-out of drug. The results represent the mean of 6 different recordings from 6 mice. * Significantly different from baseline *p* < 0.05.

## Data Availability

Data are available upon request to the corresponding author.
